# Association of admission neutrophil serine proteinases levels with the outcomes of acute ischemic stroke: a prospective cohort study

**DOI:** 10.1186/s12974-023-02758-1

**Published:** 2023-03-11

**Authors:** Lingzhi Li, Ziping Han, Rongliang Wang, Junfen Fan, Yangmin Zheng, Yuyou Huang, Zhenhong Yang, Feng Yan, Ping Liu, Haiping Zhao, Qingfeng Ma, Yumin Luo

**Affiliations:** 1 Institute of Cerebrovascular Diseases Research and Department of Neurology, Xuanwu Hospital of Capital Medical University, 45 Changchun Street, Beijing, 100053 China; 2 Beijing Key Laboratory of Translational Medicine for Cerebrovascular Diseases, Beijing, China; 3 Beijing Institute for Brain Disorders, Beijing, China

**Keywords:** Acute ischemic stroke, Neutrophil elastase, Cathepsin G, Proteinase 3, Outcome, Recombinant tissue plasminogen activator

## Abstract

**Background:**

Neutrophil serine proteinases (NSPs), released by activated neutrophils, are key proteins involved in the pathophysiologic processes of stroke. NSPs are also implicated in the process and response of thrombolysis. This study aimed to analyze three NSPs (neutrophil elastase, cathepsin G, and proteinase 3) in relation to acute ischemic stroke (AIS) outcomes and in relation to the outcomes of patients treated with intravenous recombinant tissue plasminogen activator (IV-rtPA).

**Methods:**

Among 736 patients prospectively recruited at the stroke center from 2018 to 2019, 342 patients diagnosed with confirmed AIS were included. Plasma neutrophil elastase (NE), cathepsin G (CTSG), and proteinase 3 (PR3) concentrations were measured on admission. The primary endpoint was unfavorable outcome defined as modified Rankin Scale score 3–6 at 3 months, and the secondary endpoints were symptomatic intracerebral hemorrhage (sICH) within 48 h, and mortality within 3 months. In the subgroup of patients who received IV-rtPA, post-thrombolysis early neurological improvement (ENI) (defined as National Institutes of Health Stroke Scale score = 0 or decrease of ≥ 4 within 24 h after thrombolysis) was also included as the secondary endpoint. Univariate and multivariate logistic regression analyses were performed to evaluate the association between NSPs levels and AIS outcomes.

**Results:**

Higher NE and PR3 plasma levels were associated with the 3-month mortality and 3-month unfavorable outcome. Higher NE plasma levels were also associated with the risk of sICH after AIS. After adjusting for potential confounders, plasma NE level > 229.56 ng/mL (odds ratio [OR] = 4.478 [2.344–8.554]) and PR3 > 388.77 ng/mL (OR = 2.805 [1.504–5.231]) independently predicted the 3-month unfavorable outcome. Regarding rtPA treatment, patients with NE plasma concentration > 177.22 ng/mL (OR = 8.931 [2.330–34.238]) or PR3 > 388.77 ng/mL (OR = 4.275 [1.045–17.491]) were over 4 times more likely to suffer unfavorable outcomes after rtPA treatment. The addition of NE and PR3 to clinical predictors of unfavorable functional outcome after AIS and the outcome after rtPA treatment improved discrimination as well as reclassification (integrated discrimination improvement = 8.2% and 18.1%, continuous net reclassification improvement = 100.0% and 91.8%, respectively).

**Conclusions:**

Plasma NE and PR3 are novel and independent predictors of 3-month functional outcomes after AIS. Plasma NE and PR3 also possess predictive value to identify patients with unfavorable outcomes after rtPA treatment. NE is probably an important mediator of the effects of neutrophils on stroke outcomes, which worth further investigation.

**Supplementary Information:**

The online version contains supplementary material available at 10.1186/s12974-023-02758-1.

## Introduction

Inflammation and immune response strongly contribute to ischemia-elicited tissue damage in stroke, especially causing a second insult during reperfusion [1–3]. Neutrophils are activated and recruited early to the ischemic sites by damage-associated molecule patterns (DAMPs) and show increased proteolytic activity, participating in shaping the onset, progression, and outcomes of ischemic stroke (IS) [4–6]. Neutrophil serine proteases [NSPs; neutrophil elastase (NE), cathepsin G (CTSG), and proteinase 3 (PR3)], three proteolytic effectors released from activated neutrophils [7], are involved in the pathophysiologic processes of IS [8].

A strong association between NE and cardiovascular incidents was found in a large cross-sectional clinical study that enrolled 1592 patients [9]. Furthermore, a recent clinical study enrolled 41 acute IS (AIS) patients with less than 6 h from stroke onset and matched healthy controls reported that plasma NE levels were significantly increased in AIS patients compared to controls [10], which preliminarily demonstrated that NE levels were associated with stroke severity. Serum PR3 levels were also found higher in 120 AIS patients than in matched controls [11]. Preclinical studies reported that NSPs (NE, CTSG, and PR3) augmented cerebral inflammation, enhanced vascular permeability, and caused tissue damage in experimental stroke models [12–14], which suggests that NSPs are reasonable intervention candidates in IS. However, there’s no evidence demonstrating the link between the NSPs plasma levels and the outcomes of AIS.

Recent lines of clinical studies showed that neutrophils and neutrophil extracellular traps (NETs) influence the efficacy, safety, and outcomes of recombinant tissue-type plasminogen activator (rtPA) treatment in AIS [15], by causing physical obstruction-induced no-reflow [16], reperfusion resistance [17], and hemorrhagic transformation [18]. In line with these, fundamental research suggested that NE, CTSG, and PR3 mediated the histone H3 proteolytic cleavage [19] and promoted nuclear de-condensation [20], regulating the formation of NETs. And NE was also reported extensively existed throughout all 68 thrombi retrieved from IS patients who underwent endovascular treatment [21], which indicates that NE may participate in resistance to thrombolysis. Besides, NSPs degraded basal membrane and recruited neutrophils, paving the way for neutrophils diapedesis during reperfusion in experimental stroke [12, 22]. A preclinical study reported that an NE inhibitor could reduce ischemia/reperfusion (I/R) injury in a canine heart transplantation model [23]. It’s intriguing and urgent to investigate the association between NSPs plasma levels and the outcomes of patients who received rtPA treatment.

The aim of this study, therefore, was to investigate the association between levels of NSPs and stroke outcomes as assessed by symptomatic intracerebral hemorrhage (sICH), mortality within 3 months, and modified Rankin Scale (mRS) at 3 months, in AIS patients and in patients received rtPA treatment.

## Methods

### Study design and participants

This prospective cohort study was conducted from November 2018 to November 2019. Consecutive patients diagnosed with ischemic stroke who presented within 24 h after symptom onset were enrolled in Xuanwu Hospital of Capital Medical University.

The inclusion criteria were as follows: age ≥ 18 years; a diagnosis of AIS confirmed by head computed tomography (CT) or brain magnetic resonance imaging (MRI) (hypodensity on CT; or hypo-intensity on T1-weighted imaging (T_1_WI), hyperintensity on T_2_WI and restricted diffusion on diffusion-weighted imaging (DWI)); and presentation within 24 h after symptoms onset (defined according to the “last known normal time” principle).

**Fig. 1:**
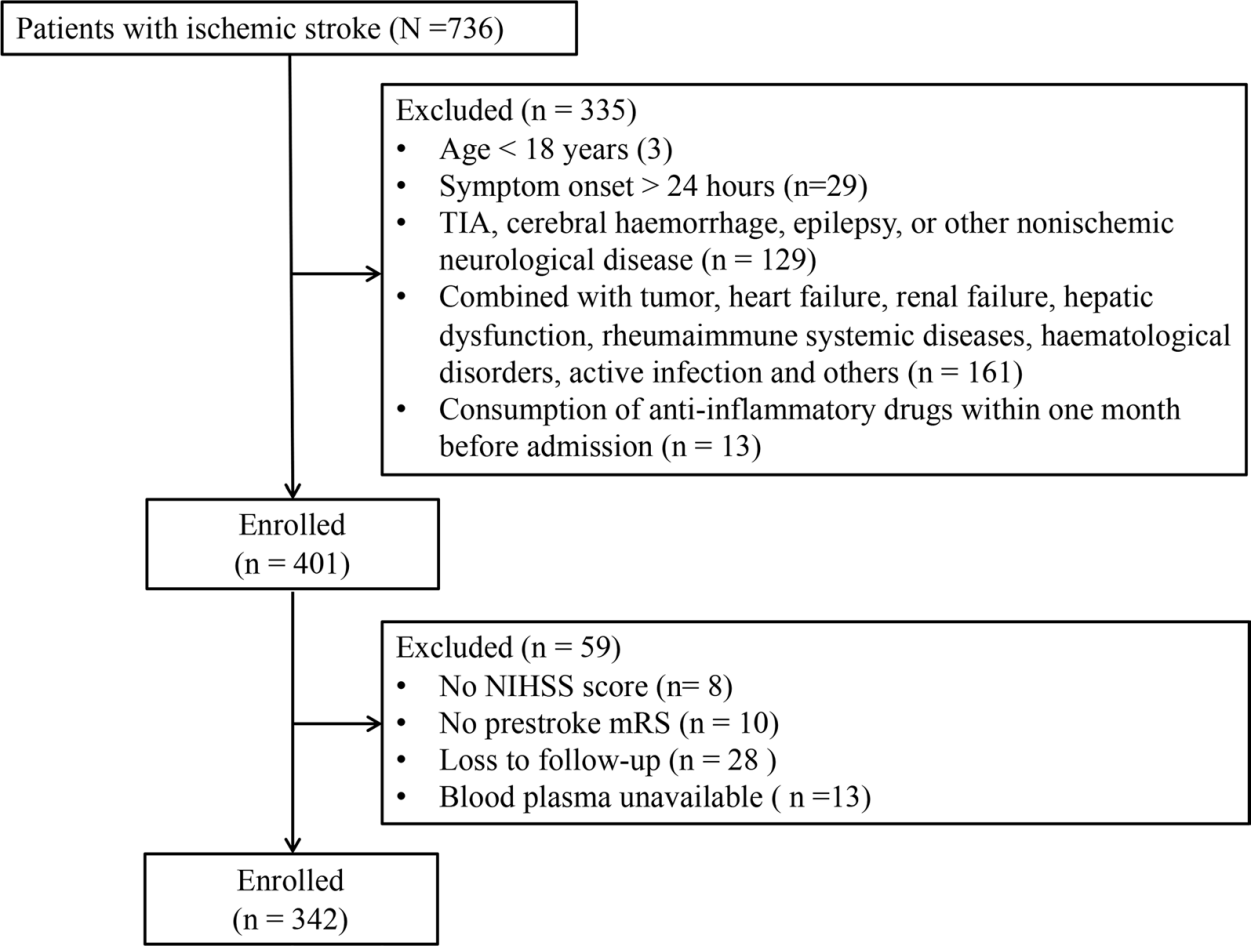
Flow chart of the study

The exclusion criteria included: TIA, cerebral hemorrhage diagnosed by baseline head CT or MRI, epilepsy, or other nonischemic neurological diseases; malignancy, heart failure (New York Heart Association class III and IV), renal failure (serum creatinine 1.5 times higher than the normal value), severe hepatic dysfunction (serum alanine aminotransferase or aspartate transaminase 2 times higher than the normal value), hematological disorders (anemia), rheumatic-immune systemic diseases and active infection; treatment with anti-inflammatory drugs within 1 month before admission. Finally, 342 AIS patients were enrolled in our analysis (Fig. 1), including 146 patients who received intravenous thrombolysis and 57 patients who received endovascular treatment.

### Clinical assessment

Baseline variables included demographic characteristics, medical history, stroke characteristics and treatment, stroke etiology, lesion location, and clinical and laboratory findings were collected from clinical interviews and neurologic examinations by board-certified neurologists. Stroke severity was assessed by the NIH Stroke Scale (NIHSS) [24] on admission in all enrolled patients. Onset-to-treatment time was defined as the time from symptom onset to admission in the emergency department. Stroke etiology was defined according to the Trial of ORG 10172 in Acute Stroke Treatment (TOAST) classification [25]. In the subgroup of patients who received rtPA treatment, NIHSS was additionally assessed within 24 h after the administration of IV-rtPA in patients, and post-thrombolysis early neurological improvement (ENI) was defined as an NIHSS score of 0 or NIHSS score decrease of ≥ 4 within 24 h [26]. The functional outcome was evaluated with modified Rankin Scale (mRS) [27] either at a face-to-face visit or by a telephone interview at 3 months by trained neurologists. We defined a favorable outcome as an mRS score of 0–2 or equal to pre-stroke mRS and an unfavorable outcome as an mRS score of 3–6. sICH was defined according to the ECASS-III [28]. All patients and investigators involved in the present study were blinded to the results of the biochemical tests.

### Outcomes

The primary study endpoint was unfavorable outcome defined as mRS 3–6 at 3 months. The secondary endpoints were sICH within 48 h and mortality within 3 months. In the subgroup of patients who received IV-rtPA, the primary study endpoint was the unfavorable outcome, and the secondary endpoints were ENI within 24 h, sICH within 48 h, and mortality within 3 months.

### Imaging

All patients underwent either a non-contrast CT or MRI scan in the emergency room. Patients who received recanalization treatment underwent another CT or MRI scan 24–36 h after treatment, or earlier in case of clinical worsening. A subgroup of patients underwent MRI within 24 h of admission (n = 212), and infarct volumes were calculated on DWI sequences as described previously [29].

### Intravenous thrombolysis administration

IV-rtPA (Actilyse; Boehringer, Ingelheim, Germany) administration was performed following the recommendations of the European stroke organization [30]. (i.e., 0.9 mg/kg body weight, maximum 90 mg, 10% of the dose as a bolus, followed by a 60-min infusion).

### Neutrophil serine proteinases measurements

All patients in the emergency department underwent blood sampling as soon as possible on admission (within 30 min from admission and within 24 h from the “last known normal time”), before IV-rtPA administration (onset-to-sampling time was nearly equal to onset-to-treatment time). Whole-blood samples were immediately centrifuged (3000 rpm, for 10 min) into plasma, then separated into corresponding vials, and stored in the − 80 °C refrigerator until analysis. All blood samples were handled complying with the same protocol throughout the whole study.

The concentrations of plasma NSPs were measured using the quantitative competitive sandwich enzyme-linked immunosorbent assay (ELISA). ELISA kit for human neutrophil elastase (cat.no. SEKH-0513) was purchased from Beijing Solarbio Science & Technology Co., Ltd, the intra-assay coefficients of variation was 6.8%, and the inter-assay coefficients of variation was 7.8%. ELISA kits for human cathepsin G and human proteinase 3 (cat.no. ER3191, ER3188) were purchased from Shanghai Guangrui Biotechnology Co., Ltd, intra-assay coefficients of variation were 6.7% and 6.3%, and the inter-assay coefficients of variation was 8.8% and 8.50%, respectively. All standards and samples were tested by board-certified laboratory technicians blinded to the clinical data, according to the manufacturers’ protocols on the same day.

### Statistical analysis

All the data were analyzed with SPSS 26 (SPSS Inc, Chicago, IL), R studio 3.6.1 (Boston, MA), and GraphPad Prism 8.4.0 (GraphPad Software, La Jolla, CA). Statistical significance was set at *p* < 0.05. The normality of data distribution was assessed with the Kolmogorov–Smirnov test. Continuous variables were described as the means (SD) in case of normal distribution or medians [IQRs] otherwise. Categorical variables were described as proportions. We analyzed the groups for continuous variables using the Student t-test or Mann–Whitney U test; and for categorical variables using the Pearson χ^2^ test or Fisher exact test, depending on the nature of data distributions.

We evaluated the association between NSPs plasma concentrations and neutrophil count and neutrophil-to-lymphocyte ratio (NLR) with spearman correlation coefficient. The predictive value of NSPs for stroke outcome was calculated with the receiver operating characteristics (ROC) curve. NE and PR3 were respectively cut off by the Youden index [31] into dichotomous variables in the whole study population and in the subgroup of patients received rtPA treatment (Additional file 3: Table S3). Then, we assessed the association of the dichotomous NE and PR3 with stroke outcome by multivariate logistic regression analysis. We entered baseline clinical variables with *p* < 0.05 (atrial fibrillation, admission NIHSS score, onset-to-treatment time, intravenous thrombolysis, endovascular treatment, SVO stroke, serum glucose, triglyceride, and neutrophil-to-lymphocyte ratio) and confounding factors (sex and age) confirmed by previous studies [32, 33] in the logistic analysis. Cox & Snell method was used to calculate the* R*^2^. Adjusted odds ratios (ORs) and related 95% confidence intervals (CIs) for each variable were calculated in the models. The crude ORs for NE or PR3 were calculated as well. The performance of models with and without NSPs for identifying patients with unfavorable functional was calculated from a 2 × 2 table using sensitivity, specificity, positive and negative predictive values, positive and negative likelihood ratios, and accuracy (Additional file 3: Table S1).

Accuracy of the predictive models with and without NSPs was further compared by calculating the areas under the curve (AUC) with the DeLong method [34]. We also quantified the improvement of discrimination and reclassification in the predictive models with the addition of NSPs by the integrated discrimination improvement (IDI) and categorical or continuous net reclassification improvement (NRI) indexes [35]. Regarding choosing the NLR or neutrophil count (for avoiding collinearity), we build the model with the NLR or neutrophil respectively (Additional file 3: Table S1). We also add the infarct size as the neuroimaging parameter into the logistic analysis in the subgroup with available infarct volume (Additional file 3: Table S5).

## Results

### Patient characteristics and clinical variables

Between November 2018 to November 2019, 736 consecutive patients with acute ischemic stroke were screened. A total of 401 patients met the inclusion criteria. Among those, 8 patients were excluded from the final analysis because of missing NIHSS scores, 10 patients for no prestroke mRS, 28 for loss to follow-up, and 13 for missing blood plasma results. Finally, 342 patients were included in the analysis (Fig. 1). Mean ± SD age was 64.4 ± 12.9 years, and 89 (26.0%) of the patients were female. The median NIHSS score was 6 [IQR 3–12] on admission, the median time from stroke onset to treatment was 2.9 [1.5–4.8] hours, and mRS 0–1 prestroke accounted for 79.8%. Among the 342 patients, 24 (7.0%) presented sICH, 27 (7.9%) died, and 222 (64.9%) had a favorable outcome (mRS at 3 months ≤ 2 or equal to prestroke mRS, Table 1) at 3 months. Among demographic characteristics, stroke characteristics and treatment, stroke etiology and clinical and laboratory findings (Table 1), a lower proportion of atrial fibrillation (12.6 vs 21.7%, *p* = 0.042, for patients with a favorable outcome and an unfavorable outcome, respectively), a lower admission NIHSS score (4.0 [2.0–7.0] vs 13.0 [8.0–17.0], *p* < 0.001), less onset-to-treatment time (2.7 [1.4, 4.4] vs 3.2 [1.8, 6.4], *p* = 0.020), a higher proportion of intravenous thrombolysis (47.3 vs 34.2%, p = 0.026), a lower proportion of endovascular treatment (11.3 vs 26.7%, *p* < 0.001), a higher proportion of small vessel occlusion (27.9 vs 15.0%, *p* = 0.010), a lower serum glucose level (6.8 [5.7, 8.8] vs 8.4 [6.3, 11.8], *p* = 0.002), a lower neutrophil count (4.6 [3.7, 5.9] vs 6.3 [4.4, 8.0], *p* < 0.001), a lower neutrophil-to-lymphocyte ratio (NLR) (2.6 [1.8, 4.0] vs 4.6 [2.7, 7.8], *p* < 0.001) and a higher triglyceride (1.6 [1.0, 2.6] vs 1.3 [0.8, 1.8], *p* = 0.002) were associated with a favorable functional outcome at 3 months.

**Table 1:** Baseline characteristics of the whole study population according to the modified Rankin Scale (mRS) at 3 months (n = 342)

	Total	Favorable outcome^a^	Unfavorable outcome	*p* value
	(N = 342)	(N = 222)	(N = 120)	
Demographic characteristics				
Age, y, mean (SD)	64.4 (12.9)	63.6 (12.0)	66.0 (14.3)	0.094
Female sex (%)	89 (26.0)	57 (64.0)	32 (36.0)	0.944
BMI, kg/m^2^, median [IQR]	25.4 [23.5, 27.4]	25.4 [23.3, 27.2]	25.8 [23.7, 27.4]	0.742
Medical history				
Hypertension	229 (67.0)	145 (65.3)	84 (70.0)	0.448
Diabetes mellitus	122 (35.8)	75 (33.9)	47 (39.2)	0.399
Hyperlipemia	86 (29.8)	64 (33.5)	22 (22.4)	0.070
Coronary heart disease	68 (19.9)	39 (17.6)	29 (24.2)	0.188
Atrial fibrillation	54 (15.8)	28 (12.6)	26 (21.7)	0.042^†^
Recurrent stroke	102 (37.1)	63 (35.4)	39 (40.2)	0.502
Smoking habit	89 (26.0)	64 (28.8)	25 (20.8)	0.121
Stroke characteristics and treatment				
Admission NIHSS score	6.0 [3.0, 12.0]	4.0 [2.0, 7.0]	13.0 [8.0, 17.0]	< 0.001^†^
mRS 0–1 prestroke	273 (79.8)	187 (68.5)	86 (31.5)	0.416
Onset-to-treatment time, h	2.9 [1.5, 4.8]	2.7 [1.4, 4.4]	3.2 [1.8, 6.4]	0.020^†^
Intravenous thrombolysis	146 (42.7)	105 (47.3)	41 (34.2)	0.026^†^
Endovascular treatment	57 (16.7)	25 (11.3)	32 (26.7)	< 0.001^†^
Stroke etiology (TOAST), n (%)				
Large artery atherosclerosis	173 (50.6)	109 (49.1)	64 (53.3)	0.526
Small vessel occlusion	80 (23.4)	62 (27.9)	18 (15.0)	0.010^†^
Cardioembolic	26 (7.6)	14 (6.3)	12 (10.0)	0.310
Other determined	11 (3.2)	5 (2.3)	6 (5.0)	0.292
Undetermined	52 (15.2)	32 (14.4)	20 (16.7)	0.692
Posterior circulation stroke	45 (13.2)	28 (12.6)	17 (14.2)	0.812
Clinical and laboratory findings				
Systolic blood pressure, mmHg	150.0 [140.0, 164.8]	150.0 [140.0, 162.8]	150.0 [140.0, 165.2]	0.693
Diastolic blood pressure, mmHg	85.5 [77.0, 92.0]	85.0 [77.0, 91.0]	89.0 [78.0, 93.5]	0.223
Serum glucose, mmol/L	7.2 [5.9, 10.1]	6.8 [5.7, 8.8]	8.4 [6.3, 11.8]	0.002^†^
HbA1c, %	6.0 [5.5, 7.3]	5.9 [5.5, 7.1]	6.2 [5.5, 7.4]	0.320
Neutrophils, ×1000/mm^3^	5.0 [3.9, 6.6]	4.6 [3.7, 5.9]	6.3 [4.5, 8.0]	< 0.001^†^
NLR	3.0 [2.0, 5.1]	2.6 [1.8, 4.0]	4.6 [2.7, 7.8]	< 0.001^†^
Platelet count, ×1000/mm^3^	207.0 [171.0, 244.8]	209.0 [174.0, 244.0]	201.0 [168.0, 245.0]	0.378
HYC, μmol/L	14.5 [11.5, 18.3]	14.6 [11.4, 18.4]	14.5 [11.9, 17.1]	0.621
TG, mmol/L	1.5 [1.0, 2.4]	1.6 [1.0, 2.6]	1.3 [0.8, 1.8]	0.002^†^
TC, mmol/L	4.5 [3.8, 5.4]	4.6 [3.9, 5.4]	4.4 [3.7, 5.0]	0.104
HDL, mmol/L	1.2 [1.0, 1.4]	1.2 [1.0, 1.4]	1.2 [1.0, 1.4]	0.773
LDL, mmol/L	2.7 [2.1, 3.4]	2.7 [2.0, 3.5]	2.6 [2.1, 3.2]	0.370
Biological measures				
NE, ng/mL	98.0 [58.6, 278.4]	95.2 [55.9, 214.2]	183.0 [80.2, 444.7]	< 0.001^†^
CTSG, ng/mL	212.4 [199.1, 224.1]	213.6 [199.0, 224.7]	210.4 [200.4, 223.4]	0.603
PR3, ng/mL	388.9 [366.0, 408.9]	386.3 [362.2, 407.1]	392.1 [378.2, 414.4]	< 0.001^†^

Data for continuous variables are described as mean (SD) (normally distributed variables) or as median [interquartile range] (nonnormally distributed variables), for categorical variables are described as n (%)

*BMI* body mass index, *CTSG* cathepsin G, *HbA1c* hemoglobinA1c, *HDL* high density lipoprotein, *HYC* homocysteine, *LDL* low density lipoprotein, *mRS* modified Rankin Scale, *NE* neutrophil elastase, *NIHSS* NIH Stroke Scale, *NLR* neutrophil-to-lymphocyte ratio, *PR3* proteinase 3, *rtPA* recombinant tissue plasminogen activator, *TC* Total cholesterol, *TG* triglyceride, *TOAST* Trial of ORG 10172 in Acute Stroke Treatment

^†^*p* < 0.05

^a^The favorable outcome was defined as an mRS score ≤ 2 or equal to prestroke mRS and the unfavorable outcome was defined as an mRS score > 2

### Relationship between the NSPs plasma concentrations and neutrophil counts

**Fig. 2:**
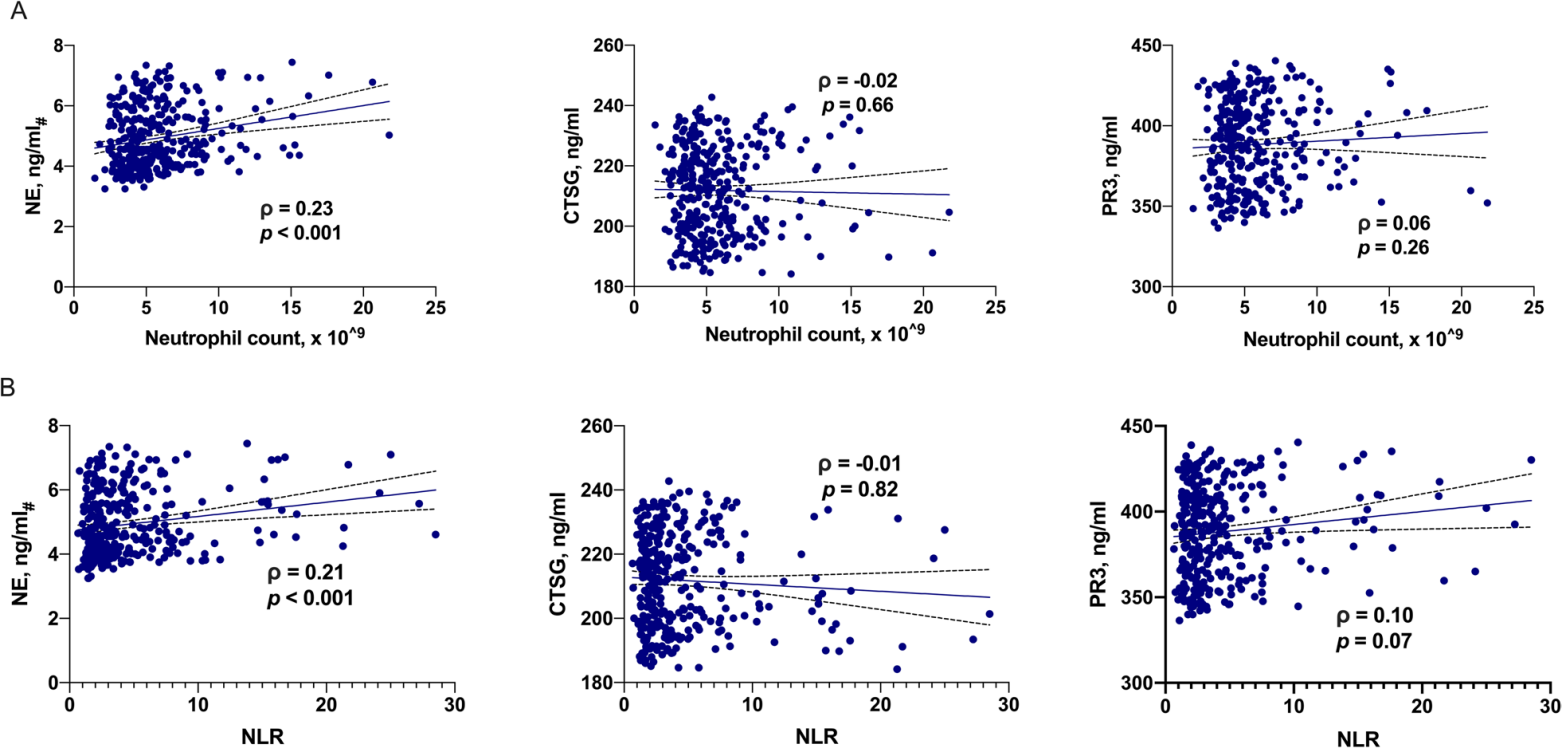
Association of plasma concentrations of neutrophil elastase (NE), cathepsin G (CTSG), and proteinase 3 (PR3) with neutrophil counts and NLR in the whole study population (n = 342). Data was analyzed by the spearman’s rank correlation, ρ represented the Spearman’s rank correlation coefficient. *NE*_*#*_ logarithmic transformation of NE plasma concentrations, *NLR* neutrophil-to-lymphocyte ratio

Increasing NE plasma concentrations were significantly correlated with increasing neutrophil counts (ρ = 0.23, *p* < 0.001) and increasing NLR (ρ = 0.21, *p* < 0.001) in the whole study population. However, CTSG and PR3 plasma concentrations were not significantly correlated with circulating neutrophil counts or NLR (Fig. 2).

In the subgroup analysis of patients with different stroke etiology, increasing NE plasma concentrations were significantly correlated with the increasing circulating neutrophil count in the large artery atherosclerosis stroke (ρ = 0.32, *p* < 0.001), but not small vessel occlusion stroke and cardioembolic stroke; PR3 plasma concentrations were negatively correlated with circulating neutrophil counts in small vessel occlusion stroke (ρ = − 0.22, *p* < 0.05) (Additional file 3: Table S2). In the subgroup analysis of patients with different lesion locations, higher NE plasma concentrations were still significantly correlated with the increasing circulating neutrophil count both in the anterior circulation stroke (ρ = 0.17, *p* = 0.003) and in the posterior circulation stroke (ρ = 0.49, *p* < 0.001).

### NE, CTSG, and PR3 levels and acute ischemic stroke outcomes

**Fig. 3:**
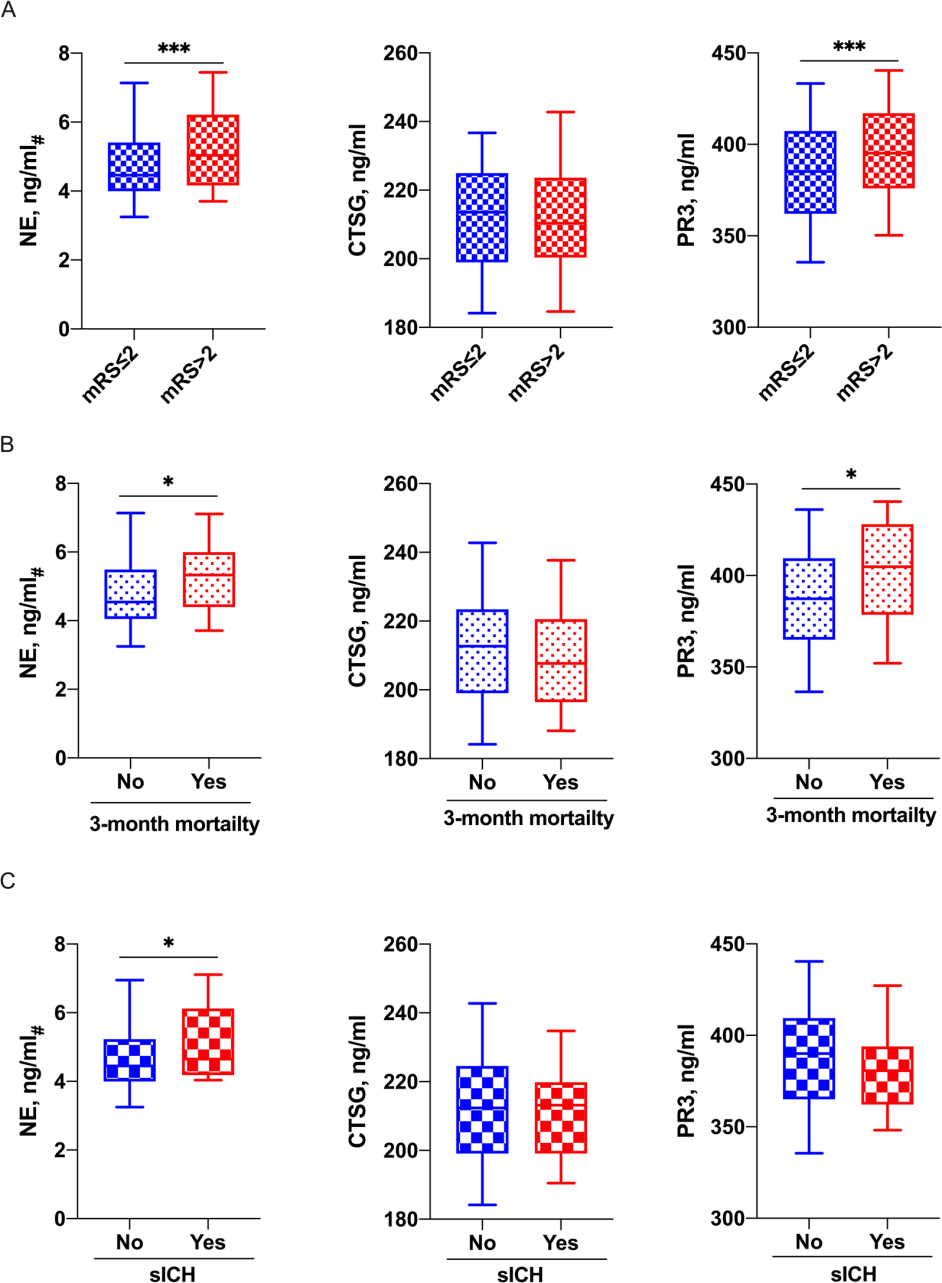
Plasma concentrations of neutrophil elastase (NE), cathepsin G (CTSG), and proteinase 3 (PR3) according to the outcomes (favorable or unfavorable outcome at 3 months, 3-month mortality, and sICH) of the whole study population (n = 342). *NE*_*#*_ logarithmic transformation of NE plasma concentrations, *mRS* modified Rankin Scale, *sICH* symptomatic intracerebral hemorrhage. ***p < 0.001; **p < 0.01; *p < 0.05

Among the three biological measures, plasma NE concentrations (95.2 [55.9, 214.2] vs 183.0 [80.2, 444.7], ng/mL, *p* < 0.001) and PR3 concentrations (386.3 [362.2, 407.1] vs 392.1 [378.2, 414.4], ng/mL, *p* < 0.001) differed significantly between patients with favorable and unfavorable outcomes at 3 months, while CTSG levels did not vary significantly (213.6 [199.0, 224.7] vs 210.4 [200.4, 223.4], ng/mL, *p* = 0.603) between the two outcomes (Table 1). When the mRS at 3 months was analyzed as a quantitative scale, mRS was positively correlated with the neutrophil counts (ρ = 0.26, *p* < 0.001), NLR (ρ = 0.28, *p* < 0.001), NE plasma concentrations (ρ = 0.22, *p* < 0.001), and PR3 concentrations (ρ = 0.17, *p* = 0.002), but the correlation was not statistically significant for CTSG plasma concentrations (ρ = − 0.08, *p* = 0.129) (Additional file 1: Fig. S1). Higher plasma NE levels were also associated with the 3-month mortality and risk of sICH, higher plasma PR3 levels were as well associated with the 3-month mortality but not the risk of sICH. CTSG plasma concentrations were not associated with any of these stroke outcome measures (Fig. 3).

### NE and PR3 predict the 3-month functional outcome after acute ischemic stroke

A cutoff point for NE of 229.56 ng/mL had 47.5% sensitivity and 77.5% specificity for the prediction of favorable functional outcome at 3 months, a cutoff point for PR3 of 388.77 ng/mL had 65.0% sensitivity and 54.5% specificity for the prediction of the unfavorable outcome at 3 months (Additional file 3: Table S3), both patients with NE plasma concentration > 229.56 ng/mL (22.5 vs 47.5%, favorable vs unfavorable outcome, *p* < 0.001) or PR3 plasma concentration > 388.77 ng/mL (45.5 vs 65.0%,* p* = 0.001) had a higher incidence of the unfavorable outcome at 3 months (Additional file 3: Table S4). Logistic regression analyses conducted confirmed plasma NE > 229.56 ng/mL (OR = 4.478 [2.344–8.554], *p* < 0.001) and PR3 > 388.77 ng/mL (OR = 2.805 [1.504–5.231], *p* = 0.001) were predictors of the unfavorable outcome at 3 months, together with admission NIHSS score (OR = 1.279 [1.196–1.368], *p* < 0.001), intravenous thrombolysis (OR = 0.393 [0.190–0.814], *p* = 0.012) and endovascular treatment (OR = 0.303 [0.118–0.779], *p* = 0.013) (Table 2). The incremental benefit of NSPs was further investigated by calculating the AUC value, the AUC for the clinical model was 86.5% and was significantly elevated to 88.9% (*p* = 0.070) when the NE and PR3 were entered into the model. It’s noteworthy that the addition of NE and PR3 to the clinical variables also improved the integrated discriminatory ability of the present predictive model (total IDI % = 8.2 [5.2, 11.2], *p* < 0.001), as well as continuous net reclassification (categorical NRI% = 12.8 [3.1, 22.6], *p* = 0.009; continuous NRI % = 100.0 [80.6, 119.4], *p* < 0.001). Detailed descriptions of logistic regression models and comparisons between the two predictive models were shown in Table 2.

**Table 2:** Logistic regression analysis and additional predictive value of the model including neutrophil elastase (NE) and proteinase 3 (PR3) concentrations for patients with unfavorable outcome (mRS > 2) at 3 months in the whole study population (n = 342)

	Unfavorable outcome^a^	
	Clinical model^b^	Clinical model^b^ + NE + PR3
Logistic regression		
R^2^ (Cox & snell)	0.326	0.385
Age	OR = 1.002 (0.978–1.026), *p* = 0.893	OR = 1.005 (0.980–1.031), *p* = 0.686
Sex (male)	OR = 1.495 (0.752–2.973), *p* = 0.252	OR = 1.789 (0.848–3.775), *p* = 0.127
Onset-to-treatment time	OR = 1.007 (0.976–1.038), *p* = 0.678	OR = 1.006 (0.964–1.049), *p* = 0.795
Admission NIHSS score	OR = 1.252 (1.177–1.331), *p* < 0.001^†^	OR = 1.279 (1.196–1.368), *p* < 0.001^†^
Atrial fibrillation	OR = 0.916 (0.327–2.568), *p* = 0.868	OR = 0.873 (0.272–2.809), *p* = 0.820
Intravenous thrombolysis	OR = 0.416 (0.210–0.825),* p* = 0.012^†^	OR = 0.393 (0.190–0.814),* p* = 0.012^†^
Endovascular treatment	OR = 0.368 (0.155–0.877), *p* = 0.024^†^	OR = 0.303 (0.118–0.779), *p* = 0.013^†^
SVO stroke	OR = 2.184 (0.618–7.718), *p* = 0.225	OR = 2.145 (0.538–8.554), *p* = 0.280
Serum glucose	OR = 1.074 (0.989–1.167), *p* = 0.090	OR = 1.074 (0.979–1.178), *p* = 0.129
Triglyceride	OR = 1.176 (0.984–1.404), *p* = 0.074	OR = 1.133 (0.920–1.397), *p* = 0.241
Neutrophil-to-lymphocyte ratio	OR = 1.080 (1.006–1.160), *p* = 0.034^†^	OR = 1.072 (0.991–1.159), *p* = 0.084
Neutrophil elastase > 229.56 ng/mL	–	OR = 4.478 (2.344–8.554), *p* < 0.001^†^
Proteinase 3 > 388.77 ng/mL	–	OR = 2.805 (1.504–5.231),* p* = 0.001^†^
ROC curve		
AUC, %	86.5	88.9
*p* Value	Ref.	0.070
IDI index, %		
Total IDI	−	8.2 (5.2, 11.2)
*p* value	Ref.	< 0.001
NRI index, %		
Categorical NRI	–	12.8 (3.1, 22.6)
*p* value	Ref.	0.009
Continuous NRI	–	100.0 (80.6, 119.4)
*p* value	Ref.	< 0.001

*AUC* area under the curve, *IDI* integrated discrimination improvement, *mRS* modified Rankin Scale, *NE* neutrophil elastase, *NIHSS* NIH Stroke Scale, *NRI* net reclassification improvement, *PR3* proteinase 3, *ROC* receiver operating characteristic curve, *SVO* small vessel occlusion

^†^*p* < 0.05

^a^The unfavorable outcome was defined as an mRS score > 2

^b^The clinical model with neutrophil-to-lymphocyte ratio for all patients with acute ischemic stroke

To test the stability of the predictive models with NE and PR3, we additionally enter the neuroimaging parameter as a covariate. In the subgroup of patients with available infarct volume (n = 212; 62.0%), after adjusting the infarct volume and clinical variables in the logistic regression for the functional outcome at 3 months, NE levels (OR = 4.641 [1.734–12.421], *p* = 0.002) and PR3 levels (OR = 3.747 [1.529–9.186], *p* = 0.004) still possessed predictive value (Additional file 3: Table S5).

### NE, CTSG, and PR3 levels and outcomes after rtPA treatment

NE, CTSG, and PR3 plasma levels did not vary significantly between patients eligible for IV-rtPA or not (Additional file 3: Table S6). Among the patients who received IV-rtPA treatment (n = 146), 27 (18.5%) patients received bridging therapy. NE, CTSG and PR3 plasma levels did not significantly alter between patients received bridging therapy or not (Additional file 3: Table S7). To analyze the association of NSPs with the outcomes of patients treated with rtPA, we excluded the patients received bridging therapy. Clinical and demographic data for the subgroup of patients received rtPA treatment (n = 119) were summarized in Additional file 3: Table S8. Favorable functional outcomes at 3 months after receiving rtPA treatment accounted for 79.0%. In the subgroup, a lower proportion of atrial fibrillation (4.3 vs 32.0%, *p* < 0.001), a lower admission NIHSS score (5.0 [3.0, 6.0] vs 9.0 [5.0, 11.0], *p* = 0.001), and a lower serum glucose on admission (6.2 [5.2, 7.7] vs 8.0 [6.1, 12.6], *p* = 0.004) were associated with a favorable functional outcome at 3 months. Among the three NSPs, higher NE plasma levels (86.7 [52.8, 199.2] vs 208.6 [69.6, 395.2], *p* = 0.041) were significantly associated with the unfavorable outcomes at 3 months, CTSG plasma levels (212.6 [198.4, 223.2] vs 215.0 [203.7, 223.3], *p* = 0.320) and PR3 plasma levels (385.6 [365.2, 404.9] vs 391.7 [378.2, 409.0], *p* = 0.170) did not differ significantly.

**Fig. 4:**
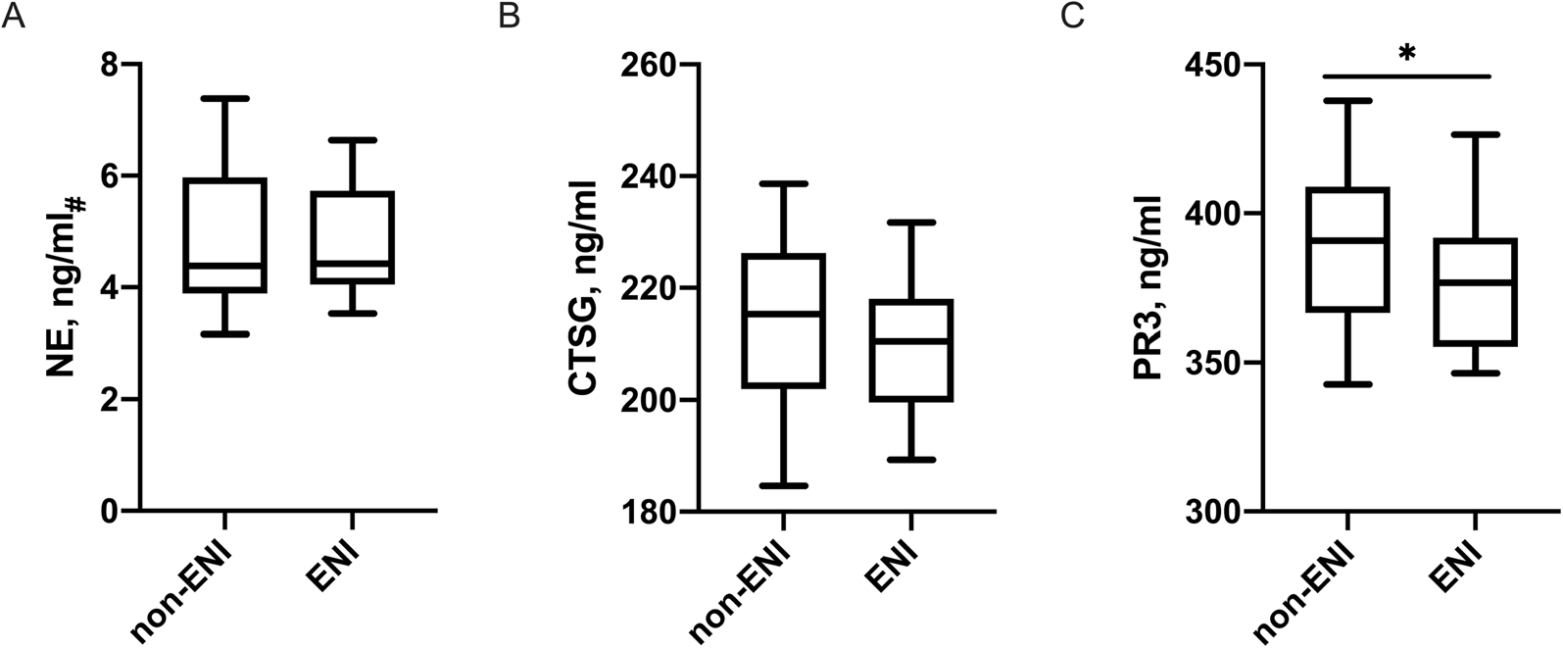
Plasma concentrations of neutrophil elastase (NE), cathepsin G (CTSG), and proteinase 3 (PR3) in different groups according to the post-thrombolysis early neurological improvement (ENI) in the subgroup of patients received rtPA treatment (n = 119). *NIHSS* NIH Stroke Scale

Within the first 24 h after rtPA treatment, 24 (21.4%) patients achieved early neurological improvement (ENI); among the three NSPs, lower PR3 plasma levels (376.6 [359.6, 391.7] vs 389.0 [366.8, 408.3], ENI vs non-ENI, *p* = 0.040) were associated with the improvement of symptoms within 24 h after rtPA treatment (Fig. 4). Besides, NE, CTSG, and PR3 plasma levels were not significantly associated with the sICH or 3-month mortality (Additional file 2: Fig. S2). In this subset, NE plasma concentrations were as well significantly correlated with circulating neutrophil counts (ρ = 0.26, *p* < 0.001) and NLR (ρ = 0.22, *p* < 0.001).

### NE predicts the 3-month functional outcome after rtPA treatment

In this subgroup of patients who received rtPA treatment, a cutoff point for NE of 177.22 ng/mL had 56.0% sensitivity and 74.5% specificity for the prediction of the unfavorable functional outcome at 3 months, a cutoff point for PR3 of 388.77 ng/mL had 68.0% sensitivity and 57.4% specificity for the prediction of unfavorable functional outcome at 3 months (Additional file 3: Table S3). Patients with NE > 177.22 ng/mL (23.4 vs 48.0%, favorable outcome vs unfavorable outcome,* p* < 0.05) or PR3 > 388.77 ng/mL (42.6 vs 68.0%, *p* < 0.05) had a higher incidence of unfavorable outcome at 3 months (Additional file 3: Table S9). Logistic regression analyses conducted in the subgroup confirmed plasma NE > 177.22 ng/mL (OR = 8.931 [2.330–34.238], *p* = 0.001) and PR3 > 388.77 ng/mL (OR = 4.275 [1.045–17.491], *p* = 0.043) were independent predictors of unfavorable outcome at 3 months, together with admission NIHSS score (OR = 1.313 [1.108–1.556], *p* = 0.002), a history of atrial fibrillation (OR = 18.824 [2.537–139.662], *p* = 0.004), and serum glucose (OR = 1.227 [1.049–1.435], *p* = 0.010). The accuracy of NE and PR3 for predicting outcome after rtPA treatment was assessed by the AUC, the clinical model was 84.6% and was lifted to 89.9% (*p* = 0.082) when the NE and PR3 were entered in the model. What’s more, addition of NE and PR3 to the clinical variables improved the integrated discriminatory ability of the present predictive model (IDI % = 18.1 [7.7, 28.5], *p* < 0.001), as well as net reclassification (categorical NRI% = 30.1 [5.3, 55.0], *p* = 0.017; continuous NRI % = 91.8 [51.1, 132.6], *p* < 0.001). Logistic regression models and comparisons between the two predictive models were described in detail in Table 3.

**Table 3:** Logistic regression analysis and additional predictive value of the model including neutrophil elastase (NE) and proteinase 3 (PR3) concentrations for unfavorable outcome (mRS > 2) at 3 months in patients received IV recombinant tissue plasminogen activator (rtPA) treatment (n = 119)

	Unfavorable outcome^a^	
	Clinical model^b^	Clinical model^b^ + NE + PR3
Logistic regression		
R^2^ (Cox & snell)	0.249	0.346
Age	OR = 0.994 (0.939–1.053), *p* = 0.843	OR = 1.018 (0.959–1.080), *p* = 0.558
Sex (male)	OR = 2.382 (0.513–11.053), *p* = 0.268	OR = 3.200 (0.588–17.425), *p* = 0.179
Onset-to-treatment time	OR = 0.706 (0.447 -1.116), *p* = 0.137	OR = 0.656 (0.373–1.154), *p* = 0.144
Admission NIHSS score	OR = 1.208 (1.064–1.371), *p* = 0.004^†^	OR = 1.313 (1.108–1.556), *p* = 0.002^†^
Atrial fibrillation	OR = 11.771 (1.981–69.947), *p* = 0.007^†^	OR = 18.824 (2.537–139.662), *p* = 0.004^†^
Serum glucose	OR = 1.159 (1.015–1.324),* p* = 0.030^†^	OR = 1.227 (1.049–1.435),* p* = 0.010^†^
Neutrophil-to-lymphocyte ratio	OR = 1.097 (0.891–1.349), *p* = 0.384	OR = 1.118 (0.896–1.395), *p* = 0.324
Neutrophil elastase > 177.22 ng/mL	Ref.	OR = 8.931 (2.330–34.238), *p* = 0.001^†^
Proteinase 3 > 388.77 ng/mL	Ref.	OR = 4.275 (1.045–17.491), *p* = 0.043^†^
ROC curve		
AUC, %	84.6	89.9
*p* value	Ref.	0.082
IDI index, %		
Total IDI	–	18.1 (7.7, 28.5)
*p* value	Ref.	< 0.001
NRI index, %		
Categorical NRI	–	30.1 (5.3, 55.0)
*p* value	Ref.	0.017
Continuous NRI	–	91.8 (51.1, 132.6)
*p* value	Ref.	< 0.001

*AUC* area under the curve, *IDI* integrated discrimination improvement, *mRS* modified Rankin Scale, *NE* neutrophil elastase, *NIHSS* NIH Stroke Scale, *NRI* net reclassification improvement, *PR3* proteinase 3, *ROC* receiver operating characteristic curve

^†^*p* < 0.05

^a^The unfavorable outcome was defined as an mRS score > 2

^b^The clinical model for the subgroup of patients received rtPA treatment

## Discussion

In the present study, we demonstrate an association between plasma NSPs levels and AIS outcomes. Patients with increased plasma NE or PR3 levels on admission are more likely to suffer an unfavorable outcome at 3 months. Of note, we find that lower PR3 plasma levels pretreatment is associated with the early neurological symptom improvement of patients who received IV-rtPA treatment, and patients receiving IV-rtPA with increased plasma NE or PR3 levels pretreatment are intended to have 3-month unfavorable outcomes than patients without. Specifically, NE and PR3 plasma levels conjointly predict the unfavorable outcomes of AIS patients, with an additional 4.478-fold risk to have an unfavorable outcome for plasma NE concentration > 229.56 ng/mL and an additional 2.805-fold risk for plasma PR3 concentration > 388.77 ng/mL after adjustment for confounders. Particularly, higher NE and PR3 plasma levels on admission also predict the 3-month unfavorable functional outcome after rtPA treatment, with an additional 8.931-fold risk to have an unfavorable functional outcome for plasma NE concentration > 177.22 ng/mL and an additional 4.275-fold risk for plasma PR3 concentration > 388.77 ng/mL after adjustment for confounders. Besides, higher plasma NE and PR3 levels are associated with 3-month mortality, and higher plasma NE levels are also associated with the risk of sICH after AIS. However, CTSG is not associated with 3-month functional outcome, 3-month mortality, or sICH after AIS. What’s more, the addition of NE and PR3 to the clinical models for predicting the 3-month unfavorable functional outcome after AIS and after rtPA treatment strongly improves the prediction efficiency.

NSPs (NE, CTSG, and PR3), the earliest formed and stored in the cytoplasmic primary (azurophilic) granules, are liberated into plasma from activated neutrophils. A study reported that serum NE level in AIS patients was significantly altered compared to controls [36]. NE was also found positive in the ipsilesional brain specimens from patients died after AIS [37]. A recent clinical study enrolled 41 AIS patients and matched healthy controls reported that plasma NE levels were significantly increased in AIS patients compared to controls, and NE levels were associated with stroke severity [10]. The knowledge of NE mediates AIS pathophysiology is strongly supported by the preclinical findings that genetic deletion or pharmacological inhibition of NE lessened blood–brain barrier (BBB) disruption, edema, and infarct volume in experimental stroke models [22, 38, 39]. Besides, NE could determine the long-term behavioral recovery from traumatic brain injury [40]. Our findings are consistent with the hypotheses from these preclinical studies.

PR3 was reported to be a direct modulator of neuroinflammation in the experimental stroke model [13]. A clinical study reported that serum PR3 level in AIS patients was significantly higher than in control subjects [11]. Higher plasma PR3 levels are also found significantly associated with AIS patients with unfavorable functional outcomes and 3-month mortality and contribute to the efficiency of the prediction model in our study, which to some extent explains the possible role of PR3 preliminarily outlined in the preclinical study [13]. No significant association between CTSG plasma levels and stroke severity, as well as outcomes has been shown in our study, though the potentially harmful role of CTSG in stroke had been indicated by pieces of preclinical findings [14, 41, 42]. Also, there is no statistical correlation has been revealed between the plasma CTSG levels and the neutrophil counts or NLR in our study. These results suggest that CTSG is probably not the main mediator of neutrophil’s contribution to stroke outcomes. Alternatively, this can be explained by the possibility that CTSG has not timely and fully responded to the DAMPs signals and degranulated from the neutrophils in the acute period of IS, for the cutoff time point in our study is the first 24 h after symptom onset.

NE partially participated in NETosis and was one component of the NETs [21, 37], which resulted in resistance to thrombolysis. Furthermore, a retrospective study that included 85 patients with anterior circulation stroke treated with thrombectomy found that higher amounts of NE-positive cells within the thrombi were independently associated with lower rates of complete recanalization [43]. Here, our findings are partially in line with it, and we observe that higher NE plasma levels pre-rtPA treatment independently predict the unfavorable functional outcome at 3 months with adjusting for cofounders. Plasma PR3 levels are associated with early neurological symptom improvement and predict the 3-month functional outcome. Though possible roles of CTSG in coupling coagulation and innate immunity have been shown in preclinical studies [44], no significant correlation between CTSG plasma concentrations and long-term outcomes has been observed in our study. Admission plasma NE and PR3 levels may thus be considered as the possible forerunners among the three NSPs in predicting the 3-month functional outcome of patients receiving rtPA treatment.

Consistent with the previous studies [10, 45, 46], we observe that neutrophil counts and NLR are associated with the stroke outcomes in our study. Whether NSPs contribute to the causal association between neutrophils and AIS outcomes? Among the three NSPs, we find that NE plasma concentrations are positively correlated with the circulating neutrophil counts in the whole study population and in the subgroup correlation analysis. A previous study reported that plasma NE was a valid inflammation marker and could be used as a parameter for the activity of neutrophils during the inflammatory response in IS [47]. The knowledge of NE mediating AIS pathophysiology is also strongly supported by the preclinical findings in experimental stroke mice [22, 37–39]. We observe higher plasma NE levels are significantly associated with sICH, mortality, and an unfavorable outcome at 3 months in AIS patients. These observations suggest that NE is probably an important mediator of the effects of neutrophils on stroke outcomes, which needs further studies.

Using predictive biomarkers associated with stroke outcomes early after stroke may help with risk stratification for stroke prognosis [5]; especially, predictive biomarkers would contribute to identifying potential treatment benefits in early clinical trials with low volume. Some neutrophils-associated prognostic markers in AIS, including MPO [48], MMP9 [48], and calprotectin [49], have been reported. MPO and calprotectin, however, are widely expressed in the myeloid line, but not exclusively in neutrophils. Compared with the two previous studies [48, 49], the findings of the three NSPs, especially the NE, in our study may be better to explain the contribution of neutrophils to the outcomes of AIS patients. From the aspect of the degranulation function of neutrophils, our study also adds preliminary evidence that supports the harmful role of neutrophils for AIS outcomes after rtPA treatment. To figure out the independent effect of the NE and PR3 on the outcomes and evaluate the predictive power of plasma NE and PR3 for the unfavorable outcome, we adjust the potential confounders, which also needs to be further confirmed by external validation in multi-cohorts of AIS patients. In the future, the two NSPs may probably aid identifying patients with unfavorable outcomes after AIS in clinical practice. For instance, given a higher NE and PR3 level may predict the unfavorable functional outcome of rtPA treatment, an AIS patient presentation with a higher NE or PR3 level might indicate the possible need for timely endovascular treatments and more intensive medical care and treatment.

Together with NE and PR3, we find stroke severity, intravenous thrombolysis, and endovascular treatment are also independent predictors of the 3-month functional outcome of all AIS patients in the present study. In the subgroup of rtPA treatment, together with NE and PR3, stroke severity, atrial fibrillation, and admission glucose level independently predict the 3-month functional outcome as well. These results are consistent with previous literature. Of note, a disproportion of gender exists in our study, male sex accounts for over two-thirds (253/342) of the whole population, which is much higher than generally expected [50]. In the present study, we find no difference between male and female in the clinical outcome after stroke, and gender disproportion does not modify the results, which is consistent with several randomized controlled trials reporting that sex did not modify treatment effect [51].

The present study should be interpreted in the context of several limitations. Firstly, from the clinical point of view, it would be interesting to further determine the statistical power of NSPs associated with the outcomes. Here, we focus on the 3-month functional outcome and test potential predictors for it but not for sICH and 3-month mortality, due to the relatively low number of events (n = 24 and 27, respectively) impeding the reliable multivariable modeling. Secondly, though we have adjusted multiple potential confounders such as sex, age, onset-to-treatment time, stroke severity, atrial fibrillation, hyperlipemia, intravenous thrombolysis, endovascular treatment, stroke etiology, serum glucose, triglyceride, and NLR in the whole cohort, effects of possible residual confounding cannot be excluded. Thirdly, the association between plasma NSPs levels and outcomes of AIS patients as well as patients received rtPA treatment, is preliminarily analyzed in a single stroke center with the limited sample size. Finally, the temporal changes of NSPs plasma levels after the onset of stroke, and both pre- and pos-rtPA treatment in AIS warrant further study.

## Conclusions

This study demonstrates higher NE and PR3 plasma levels, but not CTSG are associated with worse outcomes after AIS. Plasma NE and PR3 possess prognostic significance for the 3-month unfavorable functional outcome after AIS and possess predictive value to identify patients with the 3-month unfavorable functional outcome after rtPA treatment. The association between admission plasma NSPs levels and AIS outcomes needs to be validated in multi-cohorts and the statistical power of NSPs associated with sICH and 3-month mortality warrants to be determined. NE is probably an important mediator of the effects of neutrophils on stroke outcomes, which calls for further studies.

## Supplementary Information

**Additional file 1: Figure S1.** Association of neutrophil counts, neutrophil-to-lymphocyte ratio (NLR), and neutrophil serine proteinases with modified Rankin Scale (mRS) at 3 months in the whole study population (n = 342). CTSG, Cathepsin G; NE_#_, logarithmic transformation of neutrophil elastase plasma concentrations; NLR, neutrophil-to-lymphocyte ratio; PR3, Proteinase 3.

**Additional file 2: Figure S2.** Plasma concentrations of neutrophil elastase (NE), cathepsin G (CTSG), and proteinase 3 (PR3) in different groups according to the outcomes of patients received rtPA treatment (n = 119). rtPA, recombinant tissue plasminogen activator.

**Additional file 3: Table S1.** The performance of models with and without NSPs for identifying patients with unfavorable outcome at 3 months. **Table S2.** Bivariate correlation between neutrophil counts and neutrophil elastase (NE), cathepsin G (CTSG), as well as proteinase 3 (PR3) plasma concentrations in the subgroups. **Table S3.** The Youden index of neutrophils serine proteinases for identifying patients with unfavorable outcome at 3 months in different groups. **Table S4.** Comparisons of dichotomous neutrophil elastase (NE), and proteinase 3 (PR3) levels according to the modified Rankin Scale (mRS) at 3 months in the whole study population (n = 342). **Table S5.** Logistic regression analysis and additional predictive value of the model including neutrophil elastase (NE) and proteinase 3 (PR3) concentration for patients with unfavorable outcome (mRS > 2) at 3 months in the subgroup of patients with available infarct volume (n = 212). **Table S6.** Comparisons of neutrophil serine proteinases plasma concentrations between patients eligible for IV recombinant tissue plasminogen activator (rtPA) or not. **Table S7.** Comparisons of neutrophil serine proteinases plasma concentrations between patients received bridging therapy or not. **Table S8.** Demographic and clinical data for the subgroup of patients received IV recombinant tissue plasminogen activator (rtPA) treatment (n = 119). **Table S9.** Comparison of dichotomous neutrophil elastase (NE) and proteinase 3 (PR3) levels in the subgroup of patients received rtPA treatment according to the modified Rankin Scale (mRS) at 3 months. **Table S10.** Correlation between neutrophil serine proteinases plasma concentrations and baseline characteristics in the whole study population (n = 342).

## Data Availability

The data that support the current study are available from the corresponding author on reasonable request.

## Abbreviations

AIS: Acute ischemic stroke
NSPs: Neutrophil serine proteases
NE: Neutrophil elastase
CTSG: Cathepsin G
PR3: Proteinase 3
NLR: Neutrophil-to-lymphocyte ratio
NETs: Neutrophil extracellular traps
I/R: Ischemia/reperfusion
rtPA: Recombinant tissue-type plasminogen activator
sICH: Symptomatic intracerebral hemorrhage
mRS: Modified Rankin Scale
DAMPs: Damage-associated molecule patterns
CT: Computed tomography
MRI: Magnetic resonance imaging
NIHSS: NIH Stroke Scale
TOAST: Trial of ORG 10172 in Acute Stroke Treatment
ROC: Receiver operating characteristics
ORs: Odds ratios
CIs: Confidence intervals
AUC: Areas under the curve
IDI: Integrated discrimination improvement
NRI: Net reclassification improvement

## References

[1] Iadecola C, Buckwalter MS, Anrather J (2020). Immune responses to stroke: mechanisms, modulation, and therapeutic potential. J Clin Invest.

[2] Jayaraj RL, Azimullah S, Beiram R, Jalal FY, Rosenberg GA (2019). Neuroinflammation: friend and foe for ischemic stroke. J Neuroinflamm.

[3] Eltzschig HK, Eckle T (2011). Ischemia and reperfusion—from mechanism to translation. Nat Med.

[4] Otxoa-de-Amezaga A, Gallizioli M, Pedragosa J, Justicia C, Miró-Mur F, Salas-Perdomo A (2019). Location of neutrophils in different compartments of the damaged mouse brain after severe ischemia/reperfusion. Stroke.

[5] Lux D, Alakbarzade V, Bridge L, Clark CN, Clarke B, Zhang L (2020). The association of neutrophil-lymphocyte ratio and lymphocyte-monocyte ratio with 3-month clinical outcome after mechanical thrombectomy following stroke. J Neuroinflamm.

[6] Gong P, Liu Y, Gong Y, Chen G, Zhang X, Wang S (2021). The association of neutrophil to lymphocyte ratio, platelet to lymphocyte ratio, and lymphocyte to monocyte ratio with post-thrombolysis early neurological outcomes in patients with acute ischemic stroke. J Neuroinflamm.

[7] Stock AJ, Kasus-Jacobi A, Pereira HA (2018). The role of neutrophil granule proteins in neuroinflammation and Alzheimer’s disease. J Neuroinflamm.

[8] Jickling GC, Liu D, Ander BP, Stamova B, Zhan X, Sharp FR (2015). Targeting neutrophils in ischemic stroke: translational insights from experimental studies. J Cereb Blood Flow Metab.

[9] Tzoulaki I, Murray GD, Lee AJ, Rumley A, Lowe GD, Fowkes FG (2007). Relative value of inflammatory, hemostatic, and rheological factors for incident myocardial infarction and stroke: the Edinburgh artery study. Circulation.

[10] Weisenburger-Lile D, Dong Y, Yger M, Weisenburger G, Polara GF, Chaigneau T (2019). Harmful neutrophil subsets in patients with ischemic stroke: association with disease severity. Neurol Neuroimmunol Neuroinflamm.

[11] Elneihoum AM, Falke P, Axelsson L, Lundberg E, Lindgärde F, Ohlsson K (1996). Leukocyte activation detected by increased plasma levels of inflammatory mediators in patients with ischemic cerebrovascular diseases. Stroke.

[12] Voisin MB, Leoni G, Woodfin A, Loumagne L, Patel NS, Di Paola R (2019). Neutrophil elastase plays a non-redundant role in remodeling the venular basement membrane and neutrophil diapedesis post-ischemia/reperfusion injury. J Pathol.

[13] Cho KS, Lee EJ, Kim JN, Choi JW, Kim HY, Han SH (2015). Proteinase 3 induces neuronal cell death through microglial activation. Neurochem Res.

[14] Herrmann SM, Funke-Kaiser H, Schmidt-Petersen K, Nicaud V, Gautier-Bertrand M, Evans A (2001). Characterization of polymorphic structure of cathepsin G gene: role in cardiovascular and cerebrovascular diseases. Arterioscler Thromb Vasc Biol.

[15] Maestrini I, Strbian D, Gautier S, Haapaniemi E, Moulin S, Sairanen T (2015). Higher neutrophil counts before thrombolysis for cerebral ischemia predict worse outcomes. Neurology.

[16] El Amki M, Glück C, Binder N, Middleham W, Wyss MT, Weiss T (2020). Neutrophils obstructing brain capillaries are a major cause of no-reflow in ischemic stroke. Cell Rep.

[17] Peña-Martínez C, Durán-Laforet V, García-Culebras A, Ostos F, Hernández-Jiménez M, Bravo-Ferrer I (2019). Pharmacological modulation of neutrophil extracellular traps reverses thrombotic stroke tPA (tissue-type plasminogen activator) resistance. Stroke.

[18] Shi K, Zou M, Jia DM, Shi S, Yang X, Liu Q (2021). tPA mobilizes immune cells that exacerbate hemorrhagic transformation in stroke. Circ Res.

[19] Cheung P, Schaffert S, Chang SE, Dvorak M, Donato M, Macaubas C (2021). Repression of CTSG, ELANE and PRTN3-mediated histone H3 proteolytic cleavage promotes monocyte-to-macrophage differentiation. Nat Immunol.

[20] Papayannopoulos V, Metzler KD, Hakkim A, Zychlinsky A (2010). Neutrophil elastase and myeloperoxidase regulate the formation of neutrophil extracellular traps. J Cell Biol.

[21] Laridan E, Denorme F, Desender L, François O, Andersson T, Deckmyn H (2017). Neutrophil extracellular traps in ischemic stroke thrombi. Ann Neurol.

[22] Stowe AM, Adair-Kirk TL, Gonzales ER, Perez RS, Shah AR, Park TS (2009). Neutrophil elastase and neurovascular injury following focal stroke and reperfusion. Neurobiol Dis.

[23] Ueno M, Moriyama Y, Toda R, Yotsumoto G, Yamamoto H, Fukumoto Y (2001). Effect of a neutrophil elastase inhibitor (ONO-5046 Na) on ischemia/reperfusion injury using the left-sided heterotopic canine heart transplantation model. J Heart Lung Transplant.

[24] Lyden P, Brott T, Tilley B, Welch KM, Mascha EJ, Levine S (1994). Improved reliability of the NIH stroke scale using video training. NINDS TPA stroke study group. Stroke.

[25] Adams HP, Bendixen BH, Kappelle LJ, Biller J, Love BB, Gordon DL (1993). Classification of subtype of acute ischemic stroke. Definitions for use in a multicenter clinical trial. TOAST. Trial of Org 10172 in acute stroke treatment. Stroke.

[26] Logallo N, Novotny V, Assmus J, Kvistad CE, Alteheld L, Rønning OM (2017). Tenecteplase versus alteplase for management of acute ischaemic stroke (NOR-TEST): a phase 3, randomised, open-label, blinded endpoint trial. Lancet Neurol.

[27] Bamford JM, Sandercock PA, Warlow CP, Slattery J (1989). Interobserver agreement for the assessment of handicap in stroke patients. Stroke.

[28] Neuberger U, Möhlenbruch MA, Herweh C, Ulfert C, Bendszus M, Pfaff J (2017). Classification of bleeding events: comparison of ECASS III (European cooperative acute stroke study) and the new Heidelberg bleeding classification. Stroke.

[29] Wu C, Xue F, Lian Y, Zhang J, Wu D, Xie N (2020). Relationship between elevated plasma trimethylamine N-oxide levels and increased stroke injury. Neurology.

[30] European Stroke Organisation ESO Executive Committee, ESO Writing Committee (2008). Guidelines for management of ischaemic stroke and transient ischaemic attack 2008. Cerebrovasc Dis.

[31] Youden WJ (1950). Index for rating diagnostic tests. Cancer.

[32] Ntaios G, Papavasileiou V, Michel P, Tatlisumak T, Strbian D (2015). Predicting functional outcome and symptomatic intracranial hemorrhage in patients with acute ischemic stroke: a glimpse into the crystal ball?. Stroke.

[33] Montellano FA, Ungethüm K, Ramiro L, Nacu A, Hellwig S, Fluri F (2021). Role of blood-based biomarkers in ischemic stroke prognosis: a systematic review. Stroke.

[34] DeLong ER, DeLong DM, Clarke-Pearson DL (1988). Comparing the areas under two or more correlated receiver operating characteristic curves: a nonparametric approach. Biometrics.

[35] Pencina MJ, D'Agostino RB, Vasan RS (2010). Statistical methods for assessment of added usefulness of new biomarkers. Clin Chem Lab Med.

[36] Vogelgesang A, Lange C, Blümke L, Laage G, Rümpel S, Langner S (2017). Ischaemic stroke and the recanalization drug tissue plasminogen activator interfere with antibacterial phagocyte function. J Neuroinflamm.

[37] Denorme F, Portier I, Rustad JL, Cody MJ, de Araujo CV, Hoki C (2022). Neutrophil extracellular traps regulate ischemic stroke brain injury. J Clin Invest.

[38] Shimakura A, Kamanaka Y, Ikeda Y, Kondo K, Suzuki Y, Umemura K (2000). Neutrophil elastase inhibition reduces cerebral ischemic damage in the middle cerebral artery occlusion. Brain Res.

[39] Leinweber J, Mizurini DM, Francischetti IMB, Fleischer M, Hermann DM, Kleinschnitz C (2021). Elastase inhibitor agaphelin protects from acute ischemic stroke in mice by reducing thrombosis, blood-brain barrier damage, and inflammation. Brain Behav Immun.

[40] Semple BD, Trivedi A, Gimlin K, Noble-Haeusslein LJ (2015). Neutrophil elastase mediates acute pathogenesis and is a determinant of long-term behavioral recovery after traumatic injury to the immature brain. Neurobiol Dis.

[41] Ortega-Gomez A, Salvermoser M, Rossaint J, Pick R, Brauner J, Lemnitzer P (2016). Cathepsin G controls arterial but not venular myeloid cell recruitment. Circulation.

[42] Hooshdaran B, Kolpakov MA, Guo X, Miller SA, Wang T, Tilley DG (2017). Dual inhibition of cathepsin G and chymase reduces myocyte death and improves cardiac remodeling after myocardial ischemia reperfusion injury. Basic Res Cardiol.

[43] Kaesmacher J, Boeckh-Behrens T, Simon S, Maegerlein C, Kleine JF, Zimmer C (2017). Risk of thrombus fragmentation during endovascular stroke treatment. AJNR Am J Neuroradiol.

[44] Massberg S, Grahl L, von Bruehl ML, Manukyan D, Pfeiler S, Goosmann C (2010). Reciprocal coupling of coagulation and innate immunity via neutrophil serine proteases. Nat Med.

[45] Zhu B, Pan Y, Jing J, Meng X, Zhao X, Liu L (2018). Neutrophil counts, neutrophil ratio, and new stroke in minor ischemic stroke or TIA. Neurology.

[46] Hermann DM, Gunzer M (2019). Polymorphonuclear neutrophils play a decisive role for brain injury and neurological recovery poststroke. Stroke.

[47] Cojocaru IM, Cojocaru M, Burcin C (2006). Evaluation of granulocyte elastase as a sensitive diagnostic parameter of inflammation in first ischemic stroke. Rom J Intern Med.

[48] Maestrini I, Tagzirt M, Gautier S, Dupont A, Mendyk AM, Susen S (2020). Analysis of the association of MPO and MMP-9 with stroke severity and outcome: cohort study. Neurology.

[49] Marta-Enguita J, Navarro-Oviedo M, Rubio-Baines I, Aymerich N, Herrera M, Zandio B (2021). Association of calprotectin with other inflammatory parameters in the prediction of mortality for ischemic stroke. J Neuroinflamm.

[50] Wang M, Wang CJ, Gu HQ, Meng X, Jiang Y, Yang X (2022). Sex differences in short-term and long-term outcomes among patients with acute ischemic stroke in China. Stroke.

[51] Bushnell C, Howard VJ, Lisabeth L, Caso V, Gall S, Kleindorfer D (2018). Sex differences in the evaluation and treatment of acute ischemic stroke. Lancet Neurol.

