# Supplementary material for: Association of admission neutrophil serine proteinases levels with the outcomes of acute ischemic stroke: a prospective cohort study
Source: J Neuroinflammation. 2023 Mar 11;20:70. doi: 10.1186/s12974-023-02758-1 (PMC10007819; doi:10.1186/s12974-023-02758-1)
Supplement: Supplementary file 3 — Additional file 3: Table S1. The performance of models with and without NSPs for identifying patients with unfavorable outcome at 3 months. Table S2. Bivariate correlation between neutrophil counts and neutrophil elastase (NE), cathepsin G (CTSG), as well as proteinase 3 (PR3) plasma concentrations in the subgroups. Table S3. The Youden index of neutrophils serine proteinases for identifying patients with unfavorable outcome at 3 months in different groups. Table S4. Comparisons of dichotomous neutrophil elastase (NE), and proteinase 3 (PR3) levels according to the modified Rankin Scale (mRS) at 3 months in the whole study population (n = 342). Table S5. Logistic regression analysis and additional predictive value of the model including neutrophil elastase (NE) and proteinase 3 (PR3) concentration for patients with unfavorable outcome (mRS > 2) at 3 months in the subgroup of patients with available infarct volume (n = 212). Table S6. Comparisons of neutrophil serine proteinases plasma concentrations between patients eligible for IV recombinant tissue plasminogen activator (rtPA) or not. Table S7. Comparisons of neutrophil serine proteinases plasma concentrations between patients received bridging therapy or not. Table S8. Demographic and clinical data for the subgroup of patients received IV recombinant tissue plasminogen activator (rtPA) treatment (n = 119). Table S9. Comparison of dichotomous neutrophil elastase (NE) and proteinase 3 (PR3) levels in the subgroup of patients received rtPA treatment according to the modified Rankin Scale (mRS) at 3 months. Table S10. Correlation between neutrophil serine proteinases plasma concentrations and baseline characteristics in the whole study population (n = 342). [file 12974_2023_2758_MOESM3_ESM.docx]

**Table S1.** **The** **performance of models with and without NSPs for identifying patients with unfavorable outcome at 3 months.**

| **Characteristic** | **Sensitivity，%** | **Specificity，%** | **Positive predictive value, %** | **Negative predictive value, %** | **Accuracy, %** | **Positive likelihood ratio** | **Negative likelihood ratio** |
| --- | --- | --- | --- | --- | --- | --- | --- |
| **Model 1†** | 61.7 | 90.5 | 77.9 | 81.4 | 80.4 | 6.49 | 0.42 |
| **Model 1†** **+ NE + PR3** | 68.3 | 92.3 | 82.8 | 84.4 | 83.9 | 8.87 | 0.34 |
| **Model 2‡** | 60.8 | 89.65 | 76.0 | 80.9 | 79.5 | 5.87 | 0.44 |
| **Model 2‡ + NE + PR3** | 66.7 | 93.2 | 84.2 | 83.8 | 83.9 | 9.81 | 0.36 |
| **Model 3§** | 32.0 | 93.6 | 57.1 | 83.8 | 80.7 | 5.00 | 0.73 |
| **Model 3§ +NE + PR3** | 56.0 | 94.7 | 73.7 | 94.7 | 86.6 | 10.6 | 0.46 |
| **Model 4⊥** | 65.3 | 92.0 | 81.7 | 82.9 | 82.5 | 8.16 | 0.38 |
| **Model 4⊥+ NE + PR3** | 69.3 | 92.0 | 82.5 | 84.6 | 84.0 | 8.66 | 0.33 |

† Model 1: (age, sex, onset-to-treatment time, admission NIHSS, atrial fibrillation, intravenous thrombolysis, endovascular treatment, small vessel occlusion stroke, serum glucose, triglyceride, and neutrophil), multivariate logistic regression model for all patients with acute ischemic stroke.

‡ Model 2: (age, sex, onset-to-treatment time, admission NIHSS, atrial fibrillation, intravenous thrombolysis, endovascular treatment, small vessel occlusion stroke, serum glucose, triglyceride, and neutrophil-to-lymphocyte ratio), multivariate logistic regression model for all patients with acute ischemic stroke presented in Table 2.

§ Model 3: (age, sex, onset-to-treatment time, admission NIHSS, atrial fibrillation, serum glucose, and neutrophil-to-lymphocyte ratio), multivariate logistic regression model for the subgroup of patients received rtPA treatment presented in Table 3.

⊥ Model 4：((age, sex, onset-to-treatment time, admission NIHSS, atrial fibrillation, intravenous thrombolysis, endovascular treatment, small vessel occlusion stroke, serum glucose, triglyceride, neutrophil-to-lymphocyte ratio, and infarct volume), multivariate logistic regression model for all patients with acute ischemic stroke presented in Table e5.

**Table S2 Bivariate correlation between neutrophil counts and neutrophil elastase (NE), cathepsin G (CTSG), as well as proteinase 3 (PR3) plasma concentrations in the subgroups.**

| **Subgroups** |  | **Neutrophil count** | |
| --- | --- | --- | --- |
|  |  | **ρ** | ***p* Value** |
| **LVO stroke**  **(n = 173)** | **NE, ng/ml** | 0.32 † | <0.001 |
|  | **CTSG, ng/ml** | 0.02 | 0.779 |
|  | **PR3, ng/ml** | 0.12 | 0.110 |
| **SVO stroke**  **(n = 80)** | **NE, ng/ml** | 0.18 | 0.113 |
|  | **CTSG, ng/ml** | -0.16 | 0.149 |
|  | **PR3, ng/ml** | -0.22 † | 0.048 |
| **CEO stroke**  **(n = 26)** | **NE, ng/ml** | -0.15 | 0.478 |
|  | **CTSG, ng/ml** | -0.04 | 0.851 |
|  | **PR3, ng/ml** | 0.28 | 0.173 |
| **Anterior circulation stroke**  **(n = 297)** | **NE, ng/ml** | 0.17 † | 0.003 |
|  | **CTSG, ng/ml** | -0.05 | 0.416 |
|  | **PR3, ng/ml** | 0.07 | 0.249 |
| **Posterior circulation stroke**  **(n = 45)** | **NE, ng/ml** | 0.49 † | <0.001 |
|  | **CTSG, ng/ml** | 0.11 | 0.481 |
|  | **PR3, ng/ml** | -0.09 | 0.575 |

Abbreviations: CEO = Cardioembolic; CTSG = Cathepsin G; LVO = Large artery atherosclerosis; NE = Neutrophil elastase; NLR = Neutrophil-to-Lymphocyte Ratio; PR3 = Proteinase 3; SVO = Small vessel occlusion.

† *p* < 0.05.

**Table S3.** **The Youden index of neutrophils serine proteinases for identifying patients with unfavorable outcome at 3 months in different groups.**

|  |  | **Youden index, %** | **Cutoff point** | **Sensitivity, %** | **Specificity, %** |
| --- | --- | --- | --- | --- | --- |
| **Group 1†**  **(n = 342)** | **NE** | 25.0 | 229.56 | 47.5 | 77.5 |
|  | **PR3** | 19.5 | 388.77 | 65.0 | 54.5 |
| **Group 2**‡  **(n = 119)** | **NE** | 30.5 | 177.22 | 56.0 | 74.5 |
|  | **PR3** | 25.4 | 388.77 | 68.0 | 57.4 |

**†** Group 1: The whole study population (n = 342).

‡ Group 2: The subgroup of patients received rtPA treatment (n = 119).

**Table S4 Comparisons of dichotomous neutrophil elastase (NE),** **and proteinase 3 (PR3) levels according to the modified Rankin Scale (mRS) at 3 months in the whole study population (n = 342).**

|  | **Total** | **Favorable outcome** | **Unfavorable outcome** | ***p* Value** |
| --- | --- | --- | --- | --- |
|  | **(N=342)** | **(N=222)** | **(N=120)** |  |
| **NE > 229.56 ng/mL** | 107 (31.3) | 50 (22.5) | 57 (47.5) | < 0.001 |
| **PR3 > 388.77 ng/ml** | 179 (52.3) | 101 (45.5) | 78 (65.0) | 0.001 |

**Table** **S5. Logistic regression analysis and additional predictive value of the model including neutrophil elastase (NE) and proteinase 3 (PR3) concentration for patients with unfavorable outcome** **(mRS > 2) at 3 months** **in the subgroup of patients with available infarct volume (n = 212).**

|  | **Unfavorable outcome** | |
| --- | --- | --- |
|  | **Clinical model** ⊥ | **Clinical model** ⊥ **+** **NE + PR3** |
| **Logistic regression** |  |  |
| **R^2^ (Cox & snell)** | 0.400 | 0.446 |
| **Age** | OR = 1.030 (0.995-1.065), *p* = 0.092 | OR = 1.041 (1.004-1.080), *p* = 0.029 |
| **Sex (male)** | OR = 2.613 (0.945-7.223), *p* = 0.064 | OR = 3.408 (1.146-10.133), *p* = 0.027 |
| **Onset-to-treatment time** | OR = 1.011 (0.967-1.057), *p* = 0.630 | OR = 1.015 (0.960-1.072), *p* = 0.605 |
| **Admission NIHSS score** | OR = 1.290 (1.177-1.414), *p* < 0.001 † | OR = 1.316 (1.193-1.451), *p* <0.001 † |
| **Atrial fibrillation** | OR = 0.867 (0.251-2.993), *p* = 0.822 | OR = 1.042 (0.261-4.169), *p* = 0.953 |
| **Intravenous thrombolysis** | OR = 0.523 (0.229-1.196), *p* = 0.125 | OR = 0.491 (0.202-1.192), *p* = 0.116 |
| **Endovascular treatment** | OR = 0.299 (0.102-0.875), *p* = 0.027 † | OR = 0.247 (0.077-0.793), *p* = 0.019 † |
| **SVO stroke** | OR = 1.627 (0.315-8.403), *p* = 0.561 | OR = 1.603 (0.286-8.981), *p* = 0.591 |
| **Serum glucose** | OR = 1.063 (0.944-1.198), *p* = 0.315 | OR = 1.079 (0.948-1.227), *p* = 0.250 |
| **Triglyceride** | OR = 1.273 (1.021-1.587), *p* = 0.032 † | OR = 1.319 (1.033-1.683), *p* = 0.026 † |
| **Neutrophil-to-lymphocyte ratio** | OR = 1.124 (1.022-1.235), *p* = 0.016 † | OR = 1.110 (1.001-1.231), *p* = 0.048 † |
| **Infarct volume** | OR = 1.011 (1.002-1.020), *p* = 0.020 † | OR = 1.012 (1.002-1.022), *p* = 0.024 † |
| **Neutrophil elastase > 134.97 ng/ml** | Ref. | OR = 4.641 (1.734-12.421), *p* = 0.002 † |
| **Proteinase 3 > 388.32 ng/ml** | Ref. | OR = 3.747 (1.529-9.186), *p* = 0.004 † |
| **ROC curve** |  |  |
| **AUC, %** | 89.8 | 91.4 |
| ***p* Value** | Ref. | 0.180 |
| **IDI index, %** |  |  |
| **Total IDI** | - | 6.4 (2.8, 10.1) |
| ***p* Value** | Ref. | <0.001 |
| **NRI index, %** |  |  |
| **Categorical NRI** | - | 9.0 (-2.5, 20.5) |
| ***p* Value** | Ref. | 0.126 |
| **Continuous NRI** | - | 105.5 (82.5, 128.5) |
| ***p* Value** | Ref. | <0.001 |

* The unfavorable outcome was defined as an mRS score > 2.

† *p* < 0.05.

⊥ The clinical model with additional infarct volume for all patients with acute ischemic stroke.

Abbreviations: AUC = Area under the curve; IDI = Integrated discrimination improvement; mRS = modified Rankin Scale; NE = Neutrophil elastase; NIHSS = NIH Stroke Scale; NRI = Net reclassification improvement; PR3 = Proteinase 3; ROC = Receiver operating characteristic curve; SVO = Small vessel occlusion.

**Table S6 Comparisons of neutrophil serine proteinases plasma concentrations between patients eligible for IV recombinant tissue plasminogen activator (rtPA) or not.**

|  | **rtPA treatment** | | |
| --- | --- | --- | --- |
|  | **Yes (n = 146)** | **No (n = 196)** | ***p* Value** |
| **NE, ng/ml** | 96.0 [56.4, 261.8] | 123.8 [61.6, 286.5] | 0.056 |
| **CTSG, ng/ml** | 213.2 [199.3, 225.0] | 211.7 [199.1, 224.0] | 0.726 |
| **PR3, ng/ml** | 388.9 [367.8, 407.4] | 388.9 [365.3, 411.4] | 0.612 |

**Abbreviations:** CTSG = Cathepsin G; mRS = modified Rankin Scale; NE = Neutrophil elastase; NIHSS = NIH Stroke Scale; NRI = Net reclassification improvement; PR3 = Proteinase 3; ROC = Receiver operating characteristic curve; rtPA = recombinant tissue plasminogen activator.

**Table S7 Comparisons of neutrophil serine proteinases** **plasma concentrations between patients received bridging therapy or not.**

|  | **Bridging treatment** | | |
| --- | --- | --- | --- |
|  | **Yes (n = 27)** | **No (n = 119)** | ***p* Value** |
| **NE, ng/ml** | 97.8 [59.9, 266.6] | 94.2 [53.7, 261.6] | 0.313 |
| **CTSG, ng/ml** | 215.0 [197.2, 227.0] | 212.9 [199.6, 223.4] | 0.914 |
| **PR3, ng/ml** | 394.9 [377.5, 408.6] | 388.3 [365.4, 407.0] | 0.096 |

**Table S8 Demographic and clinical data for the subgroup of patients received IV recombinant tissue plasminogen activator (rtPA) treatment (n= 119).**

|  | **Total** | **Favorable outcome*** | | **Unfavorable outcome** | | ***p* Value** |
| --- | --- | --- | --- | --- | --- | --- |
|  | **(N=119)** | **(N=94)** | | **(N=25)** | |  |
| **Demographic characteristics** |  |  |  | |  | |
| **Age, y, median [IQR]** | 62.0 [56.0, 70.0] | 62.0 [56.0, 69.0] | | 64.0 [57.0, 73.0] | | 0.149 |
| **Male sex (%)** | 92 (77.3) | 73 (77.7) | | 19 (76.0) | | - |
| **BMI, kg/m^2^** | 25.4 [23.7, 27.5] | 25.4 [23.9, 27.6] | | 25.7 [23.5, 27.2] | | 0.874 |
| **Medical history** |  |  |  | |  | |
| **Hypertension** | 81 (68.1) | 66 (70.2) | | 15 (60.0) | | 0.464 |
| **Diabetes mellitus** | 42 (35.3) | 35 (37.2) | | 7 (28.0) | | 0.533 |
| **Hyperlipemia** | 59 (49.6) | 48 (51.1) | | 11 (44.0) | | 0.687 |
| **Coronary heart disease** | 23 (19.3) | 17 (18.1) | | 6 (24.0) | | 0.703 |
| **Atrial fibrillation** | 12 (10.1) | 4 (4.3) | | 8 (32.0) | | <0.001 † |
| **Recurrent stroke** | 37 (31.1) | 27 (28.7) | | 10 (40.0) | | 0.401 |
| **Smoking habit** | 53 (44.5) | 42 (44.7) | | 11 (44.0) | | 0.914 |
| **Stroke characteristics and treatment** | | | | | | |
| **Admission NIHSS score** | 5.0 [3.0, 7.0] | 5.0 [3.0, 6.0] | | 9.0 [5.0, 11.0] | | 0.001 † |
| **mRS 0-1 prestroke** | 112 (94.1) | 88 (93.6) | | 24 (96.0) | | - |
| **Onset-to-treatment time, h** | 2.0 [1.1, 3.3] | 2.3 [1.1, 3.5] | | 1.8 [1.4, 2.8] | | 0.527 |
| **Stroke etiology (TOAST), n (%)** | | | | | | |
| **Large artery atherosclerosis** | 69 (58.0) | 52 (55.3) | | 17 (68.0) | | 0.361 |
| **Small vessel occlusion** | 40 (33.6) | 36 (38.3) | | 4 (16.0) | | 0.063 |
| **Cardioembolic** | 7 (5.9) | 3 (3.2) | | 4 (16.0) | | 0.052 |
| **Other determined** | 1 (0.8) | 1 (1.1) | | 0 (0.0) | | - |
| **Undetermined** | 2 (1.7) | 2 (2.1) | | 0 (0.0) | | - |
| **Posterior circulation stroke** | 14 (11.8) | 10 (10.6) | | 4 (16.0) | | 0.696 |
| **Clinical and biological characteristics** | | | | | | |
| **Systolic blood pressure, mm Hg** | 152.0 [140.0, 169.5] | 150.0 [140.0, 167.0] | | 159.0 [143.0, 186.0] | | 0.140 |
| **Diastolic blood pressure, mm Hg** | 85.0 [75.0, 93.0] | 83.0 [74.0, 92.0] | | 90.0 [78.0, 97.0] | | 0.260 |
| **Serum glucose, mmol/L** | 6.5 [5.4, 8.8] | 6.2 [5.2, 7.7] | | 8.0 [6.1, 12.6] | | 0.004 † |
| **HbA1c, %** | 6.2 [5.7, 7.8] | 6.1 [5.5, 7.4] | | 6.3 [6.0, 8.7] | | 0.114 |
| **Neutrophils, ×1,000/mm^3^** | 4.6 [3.6, 5.9] | 4.6 [3.8, 5.6] | | 4.5 [3.3, 6.6] | | 0.835 |
| **NLR** | 2.5 [1.7, 4.0] | 2.4 [1.7, 3.3] | | 3.4 [2.0, 4.7] | | 0.128 |
| **Platelet count, ×1,000/mm^3^** | 215.0 [177.5, 258.0] | 215.0 [177.2, 254.0] | | 209.0 [182.0, 269.0] | | 0.982 |
| **HYC, μmol/L** | 14.1 [11.4, 18.5] | 14.0 [11.1, 18.4] | | 14.8 [13.0, 19.6] | | 0.403 |
| **TG, mmol/L** | 1.8 [1.1, 2.9] | 1.8 [1.2, 2.9] | | 1.7 [1.1, 2.4] | | 0.969 |
| **TC, mmol/L** | 4.7 [4.0, 5.6] | 4.7 [3.9, 5.6] | | 4.5 [4.1, 5.3] | | 0.837 |
| **HDL, mmol/L** | 1.1 [1.0, 1.3] | 1.1 [1.0, 1.3] | | 1.2 [1.0, 1.4] | | 0.307 |
| **LDL, mmol/L** | 2.7 [2.2, 3.5] | 2.7 [2.2, 3.5] | | 2.8 [2.4, 3.3] | | 0.557 |
| **Biological measures** |  |  |  | |  | |
| **NE, ng/ml** | 94.2 [53.7, 261.6] | 86.7 [52.8, 199.2] | | 208.6 [69.6, 395.2] | | 0.041 † |
| **CTSG, ng/ml** | 212.9 [199.6, 223.4] | 212.6 [198.4, 223.2] | | 215.0 [203.7, 223.3] | | 0.320 |
| **PR3, ng/ml** | 388.3 [365.4, 407.0] | 385.6 [365.2, 404.9] | | 391.7 [378.2, 409.0] | | 0.170 |

* The favorable outcome was defined as a mRS score ≤ 2 or equal to prestroke mRS and the unfavorable outcome was defined as an mRS score > 2.

† *p* < 0.05.

Abbreviations: BMI = Body Mass Index; CTSG = Cathepsin G; HbA1c = HemoglobinA1c; HDL = High density lipoprotein; HYC = Homocysteine; LDL = Low density lipoprotein; mRS = modified Rankin Scale; NE = Neutrophil elastase; NIHSS = NIH Stroke Scale; NLR = Neutrophil-to-Lymphocyte Ratio; PR3 = Proteinase 3; rtPA = recombinant tissue plasminogen activator; TC = Total cholesterol; TG = Triglyceride; TOAST = Trial of ORG 10172 in Acute Stroke Treatment.

Data for continuous variables are described as mean (SD) (normally distributed variables) or as median [interquartile range] (nonnormally distributed variables), for categorical variables are described as n (%).

**Table S9 Comparison of dichotomous neutrophil elastase (NE) and proteinase 3 (PR3) levels in the subgroup of patients received rtPA treatment according to the modified Rankin Scale (mRS) at 3 months.**

|  | **Total** | **Favorable outcome** | | **Unfavorable outcome** | | ***p* Value** |
| --- | --- | --- | --- | --- | --- | --- |
|  | **(N=146)** | **(N=105)** | | **(N=41)** | |  |
| **NE > 177.22 ng/mL** | 34 (28.6) | 22 (23.4) | 12 (48.0) | | 0.030 | |
| **PR3 > 388.77 ng/ml** | 57 (47.9) | 40 (42.6) | 17 (68.0) | | 0.042 | |

**Table S10 Correlation between neutrophil serine proteinases plasma concentrations and baseline characteristics in the whole study population (n = 342).**

|  | | **NE, ng/mL** | | **CTSG, ng/mL** | **PR3, ng/mL** | |
| --- | --- | --- | --- | --- | --- | --- |
| **Demographic characteristics** | |  | |  |  | |
| **Age, y** | | 0.023 | | -0.055 | 0.017 | |
| **Sex** | | -0.054 | | -0.050 | 0.074 | |
| **BMI, kg/m^2^** | | 0.036 | | -0.059 | -0.069 | |
| **Medical history** | |  | |  |  | |
| **Hypertension** | | 0.068 | | -0.020 | -0.047 | |
| **Diabetes mellitus** | | -0.039 | | 0.100 | 0.101 | |
| **Hyperlipemia** | | -0.125* | | 0.031 | -0.014 | |
| **Coronary heart disease** | | -0.004 | | -0.087 | -0.041 | |
| **Atrial fibrillation** | | 0.026 | | -0.044 | 0.004 | |
| **Recurrent stroke** | | 0.007 | | 0.034 | 0.010 | |
| **Smoking habit** | | 0.142 | | -0.146 | 0.041 | |
| **Stroke characteristics and treatment** | | | | | | |
| **Admission NIHSS score** | | 0.062 | | -0.020 | 0.037 | |
| **mRS 0-1 prestroke** | | 0.016 | | 0.064 | 0.057 | |
| **Onset-to-treatment time, h** | | 0.046 | | 0.032 | 0.016 | |
| **Intravenous thrombolysis** | | -0.103 | | 0.019 | -0.027 | |
| **Endovascular treatment** | | 0.019 | | 0.070 | 0.066 | |
| **Stroke etiology (TOAST)** | | | | | |  |
| **Large artery atherosclerosis** | 0.004 | | -0.022 | | 0.074 |  |
| **Small vessel occlusion** | -0.044 | | 0.009 | | 0.035 |  |
| **Cardioembolic** | 0.059 | | 0.042 | | -0.032 |  |
| **Other determined** | -0.051 | | 0.017 | | 0.051 |  |
| **Undetermined** | 0.028 | | -0.020 | | -0.146 |  |
| **Posterior circulation stroke** | -0.016 | | 0.044 | | -0.094 |  |
| **Clinical and laboratory findings** | | | | | |  |
| **Systolic blood pressure, mm Hg** | | 0.008 | | -0.017 | 0.038 | |
| **Diastolic blood pressure, mm Hg** | | 0.048 | | -0.023 | 0.047 | |
| **Serum glucose, mmol/L** | | 0.086 | | -0.065 | 0.111* | |
| **HbA1c, %** | | -0.043 | | -0.015 | 0.196** | |
| **Neutrophils, ×1,000/mm^3^** | | 0.217** | | -0.024 | 0.057 | |
| **NLR** | | 0.189** | | 0.016 | 0.098 | |
| **Platelet count, ×1,000/mm^3^** | | -0.008 | | -0.078 | -0.095 | |
| **HYC, μmol/L** | | -0.118 | | 0.036 | 0.004 | |
| **TG, mmol/L** | | 0.004 | | -0.036 | -0.016 | |
| **TC, mmol/L** | | 0.003 | | -0.012 | -0.004 | |
| **HDL, mmol/L** | | -0.032 | | 0.050 | -0.027 | |
| **LDL, mmol/L** | | 0.036 | | 0.003 | -0.014 | |

Abbreviations: BMI = Body Mass Index; CTSG = Cathepsin G; HbA1c = HemoglobinA1c; HDL = High density lipoprotein; HYC = Homocysteine; LDL = Low density lipoprotein; mRS = modified Rankin Scale; NE = Neutrophil elastase; NIHSS = NIH Stroke Scale; NLR = Neutrophil-to-Lymphocyte Ratio; PR3 = Proteinase 3; rtPA = recombinant tissue plasminogen activator; TC = Total cholesterol; TG = Triglyceride; TOAST = Trial of ORG 10172 in Acute Stroke Treatment.

* *p* < 0.05; ** *p* < 0.01.
